# Defoliation in mangrove saplings vary depending on species and environment

**DOI:** 10.3897/BDJ.13.e140659

**Published:** 2025-05-23

**Authors:** Karthigan Ramatas, Feiyang Wen, Mohamad Azlin bin Sani, Von Bing Yap, Eunice Jingmei Tan

**Affiliations:** 1 Division of Science, Yale-NUS College, Singapore, Singapore Division of Science, Yale-NUS College Singapore Singapore; 2 Department of Statistics and Data Science, National University of Singapore, Singapore, Singapore Department of Statistics and Data Science, National University of Singapore Singapore Singapore; 3 Department of Biological Sciences, National University of Singapore, Singapore, Singapore Department of Biological Sciences, National University of Singapore Singapore Singapore

**Keywords:** mangrove saplings, *Rhizophora*, *Bruguiera*, defoliation, aquaculture

## Abstract

As mangrove ecosystems are rapidly being degraded worldwide, it is essential to understand how we can protect them. Defoliation of mangrove saplings can negatively affect mangrove ecosystems’ health and resilience, and its effects can be further exacerbated and accelerated by habitat disturbances such as climate change and urban development. We examined the levels of defoliation across four different species of mangrove saplings—*Bruguieracylindrica*, *B.gymnorhiza*, *Rhizophoraapiculata* and *R.mucronate* across ten sites on Pulau Ubin, Singapore. We found that different species of mangrove saplings suffered different rates of defoliation, and this could be because of interaction effects from proximity to roads, river mouth and past site use for aquaculture.

## Introduction

Mangrove ecosystems are rapidly being degraded worldwide, with a 30% reduction over the past 50 years (Alongi 2002, Trisnawati et al. 2019). Urban development and climate change are major factors contributing to the fragmentation and vulnerability of mangrove habitats (Gilman et al. 2006). In Southeast Asia, which host a third of the world’s mangroves, there was a yearly decrease of 0.18% of mangrove area between 2000 and 2012, amounted to 100,000 ha lost (Richards and Friess 2015). Yet, mangroves play key roles in maintaining coastal ecosystems and communities (Danielsen et al. 2005, Kathiresan and Rajendran 2005, Othman 1994), and it is essential to understand how they can be better protected and managed. Herbivory in mangroves can negatively affect their health and resilience, and its effects can be further exacerbated and accelerated by habitat disturbances such as climate change and urban development (Friess et al. 2012, Maldonado-López et al. 2019).

Herbivory plays an indispensable role in mangrove nutrient cycling pathways (Feller 2002, Lugo and Snedaker 1974). However, high rates of insect herbivory can reduce the mangroves’ ability to photosynthesize, weak their reproductive capacity (Neveu 2013, Trisnawati et al. 2019), and cause mangrove defoliation and death (Anderson and Lee 1995, Schowalter et al. 1986). Mangrove herbivory by insect folivores can be severe (e.g. Anderson and Lee 1995, Duke 2012, Gara et al. 1990, Piyakarnchana 1981), causing high defoliation rates among mangroves, resulting in their large-scale dieback such as canopy loss of *Rhizophoramangle* forests in Belize (Feller and Mathis 2006). Insect herbivory can be especially damaging to saplings in particular, as they have fewer physical defences than mature trees such as leaf toughness (Reich et al. 1999, Wright et al. 2004, Zvereva et al. 2020), as well as being richer in chemical compounds like foliar nitrogen, making them more palatable to leaf-chewing insects (Zverev et al. 2017, Zvereva et al. 2020). Combined with the lower avian predation rates of insects on shorter plants (Zvereva et al. 2020), saplings are more vulnerable to insect herbivory than mature trees (Burrows 2003, Trisnawati et al. 2019).

The paucity of studies on mangrove herbivory could be attributed to the perceived lower rate of herbivory in mangroves compared to other forest types (Metcalfe et al. 2013), because of the presence of high concentration of chemical compounds like phenol and tannins (Kathiresan 2003), or the toughness of leaves (Roberson and Duke 2006) that deter herbivores. However, recent studies indicate that the diversity of insect fauna in mangroves and their impact on mangroves due to herbivory are greater than previously thought to be (Duke 2012, Yeo et al. 2021). Several studies highlight that mangrove herbivory in saplings is greater than in adult mangroves (Cannicci et al. 2008, Feller 2002, Feller and Mathis 2006, Zverev et al. 2017). Scale insect infestation can be deadly to *R.mucronata* saplings (Ozaki et al. 2001), while Lepidopteran folivores on *R.mangle* led to the saplings losing up to 50% of their leaf area within three months (Ellison and Farnsworth 1996). Severe defoliation can negatively affect mangrove regeneration efforts in disturbed mangroves (Elster et al. 1999). For instance, herbivory of flower buds can lead to flowers not reaching maturity, thus compromising future propagation (Anderson and Lee 1995).

Factors ranging from distance to roads and river mouths, mangrove species, plant height, rate of growth and site history can further affect the extent of herbivory on mangroves. Distance from nearby roads and motorways can affect herbivory, as plants near the road can suffer higher rates of insect herbivory (Angold 1997). Disturbed mangrove habitats show a greater defoliation rate, experiencing leaf herbivory of up to 16% more compared to undisturbed habitats (Kihia et al. 2011, Maldonado-López et al. 2019). Anthropogenic disturbances can also significantly lower substrate pH and harvestable tree abundance (Kihia et al. 2011). Fragmentation caused by roads built between the river and mangroves disrupts hydrologic connectivity, leaving mangroves dry and saline (Akram et al. 2023, Newton et al. 2020). The disruption in hydrological connectivity also results in increased sedimentation, nutrient enrichment of nitrogen and phosphorus as well as decreased habitat quality affecting organisms that live near the road (Allgeier et al. 2010, Valentine‐Rose and Layman 2011). Proximity to river mouths can influence the domination of different mangrove species through their adaptations to the different soil quality and salinity levels (Chen and Twilley 1999, He et al. 2021). Areas close to the river mouth experience greater sediment trapping (Vundavilli et al. 2021), which has implications on how sediments influence the nutrient availability in mangrove soil, affecting the substrate for insect herbivores or their associated host plants (Chen and Twilley 1999). For instance, nutrients such as nitrogen and phosphorus are higher at the river mouth (Chen and Twilley 1999). The salinity gradient and the freshwater river input to the mangroves also affect the soil quality and surrounding vegetation (Bernardino et al. 2022, He et al. 2021). Mangrove species and site locations in turn affect herbivory levels (Roberson and Duke 2006), with higher herbivory on plants that are growing vigorously, and greatest in tall trees (Feller and Mathis 2006).

Changes to land-use, such as the conversion of mangroves for aquaculture is widespread and can be detrimental to mangroves. From 2000 to 2016, 62% of mangrove losses were caused by land-use changes related to aquaculture and agriculture (Friess et al. 2019, Goldberg et al. 2020). The main contributor to mangrove area decline in Southeast Asia is the expansion of aquacultural area (Yunlei Chang and Zhang 2023). Conversion of mangrove habitats for aquaculture such as shrimp farms may be particularly detrimental to mangroves, as mangrove trees were removed for the construction of ponds, and the interior of the ponds levelled (Tham 1973). Apart from changing soil infrastructure, shrimp farms can emit high amounts of nitrogen and phosphorus, causing soil damage and leading to eutrophication (de Lacerda et al. 2021, Marins et al. 2020, Primavera et al. 1993). Soil damage and eutrophication in turn affect mangrove functioning by impairing the mangrove ecosystem’s capacity to retain nutrients such as carbon (Merecí-Guamán et al. 2021, Queiroz et al. 2019). Anthropogenic stress can also initiate significant changes to the mangrove habitat, which can drive community structure and function through both top-down effects of predators and bottom-up effects of resources (Forde et al. 2022, Maldonado-López et al. 2019). Through top-down effects, avian insectivores can control arthropod abundance and subsequent herbivory in mangroves, which could influence bottom-up effects such as detrital subsidy, which can result in mangrove leaves being more vulnerable to damage (Forde et al. 2022).

Mangroves in Singapore were initially deforested for fuel and firewood, and later subject to industrialization, agriculture, aquaculture, and land reclamation. As a result, the area of mangroves has decreased massively from approximately 75km^2^ in 1819, to less than 7 km^2^ today (Yee et al. 2010). To understand the factors that can affect herbivory of mangrove saplings, we surveyed the levels of defoliation across four species – *Bruguieracylindrica*, *B.gymnorhiza*, *R.apiculata*, and *R.mucronate* across different sites on an island Pulau Ubin, Singapore. We ask if the rate of defoliation varies with the species, size class and the extent of human disturbances in terms of distance from the road and river mouth as well as site history.

## Material and methods

**Study site**

**Figure 1.**
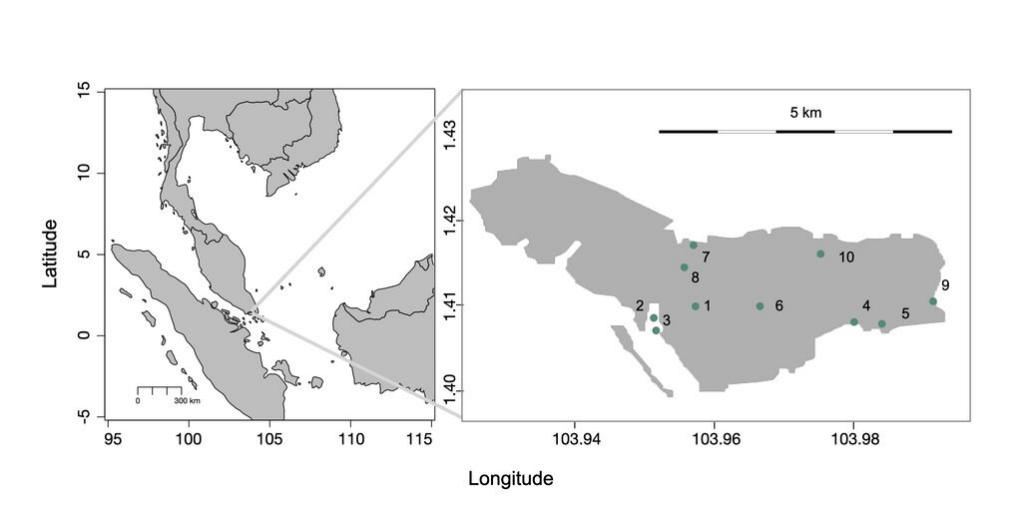
Map of Malay Peninsula and surrounds. Inset: Pulau Ubin, Singapore, with study site locations indicated by teal-coloured circles.

Defoliation rates of mangrove saplings were surveyed at Pulau Ubin (1°24′34″N 103°57′36″E), an island northeast of Singapore. The island features coastal areas with a mix of mangrove and intertidal habitats (Ng et al. 2011). Ten mangrove sites across the island were sampled to capture the variability of mangrove habitats within the island (Fig. 1).

**Defoliation assessment**

Understanding the extent of defoliation requires reliable estimates of damage. Visual estimates provide a non-destructive, yet fast and cost-effective method to quantify defoliation (Johnson et al. 2015). To ensure precision (repeatability of measurements) and accuracy (bias in estimations), observers were trained to estimate leaf damage percentages following Johnson et al. 2015. Observers spent 15-20 seconds assessing each leaf, visualizing the leaf as sections (e.g. halves, quarters) and estimating damage to each section. Multiple leaf samples with varying herbivory levels were used for training. The observers then came to a consensus on the different visual estimates of defoliation based on the varying appearance of the leaves. We identified saplings of four abundant mangrove species in Pulau Ubin: *B.cylindrica* (BRCY), *B.gymnorhiza* (BRGY), *R.apiculata* (RHAP), and R.mucronate (RHMU) and examined the level of herbivory on these saplings. Saplings were catgorized by height class (Class 1: 1.5m to 2m with 323 samples; Class 2: 2m to 2.5m with 122 samples). The highest 20 leaves from each sapling were selected, and leaf damage was visually estimated using semi-quantitative bins (0%, 1-10%, 11-25%, 26-50%, 51-75%, >75%) based on the percentage of leaf lamina area removed (Suppl. material 1).

**Effects of land use**

To understand the effects of land use on defoliation levels, we collected data on the distance of saplings from roads, past site use in aquaculture and proximity to rivers. The sites had varying distances to 1) roads with vehicular and foot traffic (Jalan Wat Siam, Jalan Durian, and Jalan Noordin); 2) biking trails (Ketam Mountain Bike Trail); and 3) pedestrian trail (Chek Jawa Trail). We used Google Maps to measure the shortest distance from each sapling to the nearest road by selecting the function 'Measure distance' on the website. Sites were scored for past aquaculture use based on the presence of man-made ponds and bunds during our surveys, and from historical records (Chia 1992, Department of Geography National University of Singapore 2023, NUS Libraries Historical Maps of Singapore)(Table 1). We also categorized site locations relative to rivers using Google Maps to consider the potential effect of proximity to freshwater.

**Data analyses**

Data analyses were conducted using R (version 4.3.1). To calculate the defoliation rate of each leaf, we used a uniform distribution adapted to the assigned range: 0%, 1-10%, 11-25%, 26-50%, 51-75%, and >75%. For example, if a leaf is in the bin 1-10%, we generated a number uniformly from 1 to 10. This procedure was applied to all leaves to derive a mean defoliation rate for each tree. We examined the effects of the following variables on defoliation rate: 1) mangrove sapling species, 2) sapling height, 3) distance from road, 4) past aquaculture history, and 5) proximity to river mouths. A linear model was built to understand how species, height, distance from road, and site history affect the defoliation rate of trees. An initial full-factorial model included all interactions, but no significant interaction was found between species and river mouth. Therefore, we analyzed a reduced model with interactions between mangrove sapling species and height, and among proximity to river mouth, distance from roads, mangrove sapling species and past aquaculture history. As the imputation procedure contains random elements, we repeated the imputation of defoliation rates 1,000 times, and looked for robust results from the regression analysis. We visually summarized the p-values associated with the variables and computed the proportion of significant effects across the 1,000 runs. The Benjamini-Hochberg procedure was applied to control for multiple comparisons, ensuring that the reported P-values reflect the true significance of the variables.

## Results

**Figure 2.**
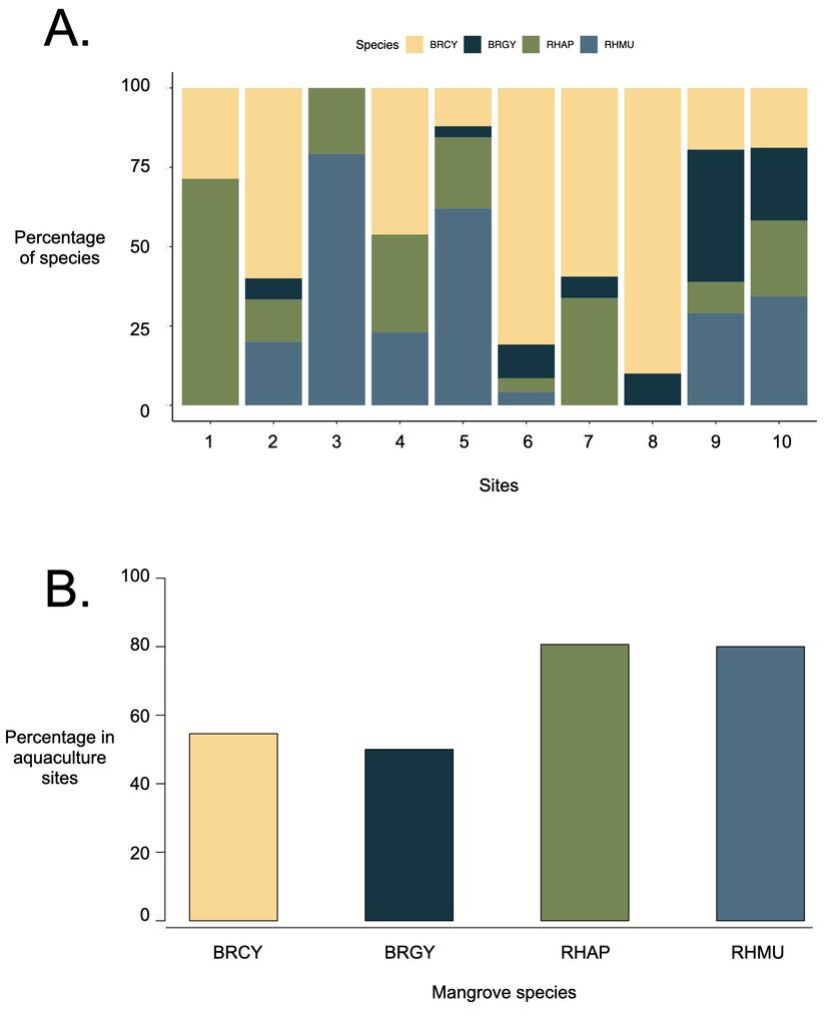
Distribution of mangrove species across A) all sites; B) only sites with known aquaculture history.

We surveyed a total of 152 BRCY saplings, 72 BRGY saplings, 93 RHAP saplings and 130 RHMU saplings across 10 sites at Pulau Ubin. While the majority of sites exhibited a presence of all four species, the specific distribution varies across sites. Some sites hosted only two or three species (Fig. 2A), while a significant number of *Rhizophora* individuals were found in former aquaculture sites (Fig. 2B).

Our initial analyses revealed that, compared to BRCY, both BRGY and RHMU are less susceptible to defoliation (estimated effect BRGY: -20.78±1.96, RHMU: -25.76±1.96, Suppl. material 1). In contrast, RHAP (88.39±11.09) shows a significantly higher defoliation rate, indicating greater vulnerability to herbivore interactions compared to BRCY and the other species. All sites, except Site 5 (-23.53±16.24, Suppl. material 3), have higher herbivory rates compared to Site 1. This may be due to site history, so we further investigated by regrouping the sites based on past aquaculture use (Suppl. material 2). We examined whether species composition was associated with the geographical characteristics of the individual plots, as well as their historical context and ecological evolution over time. Species was a significant predictor across 1,000 runs, with p-values consistently smaller than 0.05, indicating a strong and reliable influence on defoliation rate (Table 2). Variables with interaction terms involving species (Species: River mouth: Distance 0.999) also consistently showed significant effects (Suppl. materials 3, 4). Conversely, variables like sapling height class (0.105) and site (0.000) did not exhibit consistent significance. These findings highlight the importance of species as a predictor. Site and distance from roads (River mouth: Distance: Aquaculture, 0.977) consistently showed significant effects.

## Discussion

Our study found that the different species of mangrove saplings experienced varying rates of defoliation, and this could be because of interaction effects from proximity to roads, river mouths and past site use for aquaculture. In addition, the species distribution across sites varied, with certain sites hosting two species each, while sites 3 and 7 hosted three species (Figure 2A).

Differences in defoliation across mangrove species may result from varying herbivory preferences due to leaf toughness and unpalatable chemicals. Interspecific variations in herbivory exist, with some species experiencing higher levels, possibly due to lower tannin levels (Trisnawati et al. 2019). Leaf area loss varied widely from 0.3% to 35% across 25 mangrove species studied in Australia (Roberson and Duke 2006). *Rhizophora* species, which have tougher leaves, were less susceptible to herbivory and leaf area loss compared to species like *Avicenniamarina* with more succulent leaves (Roberson and Duke 2006). Species with more succulent leaves may also be more susceptible to herbivory. For instance, the caterpillars of *Junoniaevarete* only fed on the succulent *A.germinans* mangroves in Costa Rica and not other species such as *R.mangle* and *Lagunculariaracemosa*. This preferential feeding by caterpillars resulted in defoliation rates on *A.germinans* mangrove ranging between 18-50% (Elster et al. 1999). Other species of mangroves may also suffer less herbivory than others due to their chemical defences. For instance, leaves of *Excoecariaagallocha* faced less than 1% defoliation due to the toxic chemicals in its sap that deters insects from consuming them (Roberson and Duke 2006). Similarly, *Rhizophora* leaves have high phenol concentrations and high carbon:nitrogen ratios, reducing palatability to insects and decreasing defoliation rates (Maldonado-López et al. 2019, Menezes and Peixoto 2009). With up to 102 insect herbivores documented to feed on mangroves in Singapore (Murphy 1990), these insects likely have varying preferences to leaf characteristics.

Individually, factors such as proximity to roads, river mouths and past aquaculture use have no significant impact on mangrove herbivory, but their interactions played a significant role. This could be due to increased dryness and salinity of mangroves caused by fragmentation from roads (Akram et al. 2023, Newton et al. 2020). Additionally, roads contribute to sediment runoff and increase nutrients like phosphorus and nitrogen (Allgeier et al. 2010, Valentine‐Rose and Layman 2011). Aquaculture further exacerbates this issue as it similarly increases sediments and nutrients (de Lacerda et al. 2021, Marins et al. 2020, Primavera et al. 1993), and river mouths trap sedimentation, affecting mangrove soil quality (Vundavilli et al. 2021). Riverine saplings of *Bruguiera* and *Rhizophora* were shorter than saplings in other zones (Ewel and Baldwin 2022), which could be a result of higher defoliation levels at river mouths, as observed in our study. Different mangrove species will have varying adaptations to cope with changes in soil quality (Kathiresan 2011), which can then influence the composition of herbivorous communities within the mangrove ecosystem. The extent of insect herbivory may not be significantly different between forest gaps and road edges due to changes in the abundance of herbivores (Santos and Benítez‐Malvido 2011).

Conversion to aquaculture affects mangroves in several ways. During the construction of ponds for shrimp farming, mangrove fringes were left between the shore and the pond to protect the ponds against direct wave action (Tham 1973). Creating barriers to contain the cultures also alters mangrove salinity levels (Bryan-Brown et al. 2020), which can affect the host plants of insect herbivores, as some host plants are better adapted to higher salinity (Barik et al. 2017), while other host plants may reduce leaf size (Cao et al. 2023). Together, this would affect the availability of host plants to insect herbivores.

Understanding how defoliation varies across different mangrove sapling species is essential for rehabilitating, reforesting, and protecting mangroves. Defoliation can lead to reduced soil carbon losses (Servais et al. 2018), which has further implications on mangroves as carbon stores. This knowledge allows informed decisions on suitable species for reforestation projects to reduce defoliation. Investigating how defoliation rates vary between different height classes of saplings is important for analyzing how different stressors affect defoliation rates at different heights. Knowledge of these stressors will enable targeted conservation efforts to address them, reducing extreme defoliation. Finally, understanding how urban disturbances affect defoliation will help infer how roads and site history, such as previously abandoned aquaculture ponds, may change mangrove leaf chemistry, influencing insect herbivory rates.

## Supplementary Material

C49584CC-6AE9-594C-BFE3-592B3BDAC107 10.3897/BDJ.13.e140659.suppl1 Supplementary material 1 Figure S1 Data type image Brief description Violin Plot for Distribution of p-values Across Variables. This violin plot shows p-value distributions across 1,000 imputation runs for each variable. The horizontal line at p=0.05 marks the significance threshold. Plot, Plot intersect with distance, and Species consistently show significant effects (distributions mostly below 0.05), while Distance and Height remain largely non-significant. This visualization identifies which factors reliably influence defoliation rates despite randomness in the imputation process. File: oo_1270733.png https://binary.pensoft.net/file/1270733 Feiyang Wen

6B442239-B77A-55A1-A97E-E36ECB8ADC8E 10.3897/BDJ.13.e140659.suppl2 Supplementary material 2 Figure S2 Data type image Brief description Violin Plot for Distribution of p-values Across Regrouped Variables. This violin plot displays p-value distributions across 1,000 imputation runs examining factors affecting defoliation rates. The horizontal line at p=0.05 marks the significance threshold. Distance, Height, Rivermouth:Distance, and Species:Rivermouth:Distance consistently show significant effects (distributions mostly below 0.05). Species was a particularly strong predictor, with p-values consistently below 0.05 across all runs. This visualization confirms which factors reliably influence defoliation patterns when considering site history and geographical characteristics, despite randomness in the imputation process. File: oo_1270735.png https://binary.pensoft.net/file/1270735 Feiyang Wen

93DAADB5-E437-50E4-9A25-3C558F12CE60 10.3897/BDJ.13.e140659.suppl3 Supplementary material 3 Table S1 Data type table Brief description Coefficient estimates and standard errors (to 2 d.p.) from interaction effects from proximity to roads and species File: oo_1162781.docx https://binary.pensoft.net/file/1162781 Feiyang Wen

B69D274F-41F9-5BC9-A2BE-61AA0D44E1E6 10.3897/BDJ.13.e140659.suppl4 Supplementary material 4 Table S2 Data type table Brief description Coefficient estimates and standard errors (to 2 d.p.) for interaction effects from species, river mouth and past site use for aquaculture. File: oo_1162784.docx https://binary.pensoft.net/file/1162784 Feiyang Wen

## Figures and Tables

**Table 1.** Site location in relation to past aquaculture use and river mouth

Site	Past aquaculture use	River mouth
1	No	No
2	Yes	No
3	Yes	No
4	Yes	No
5	No	No
6	Yes	No
7	No	Yes
8	No	No
9	No	No
10	Yes	Yes

**Table 2.** Robustness of variables influencing sapling defoliation rate

Variable name	Proportion of P < 0.05 over 1,000 runs
Height	0.105
Species	1.000
River mouth	0.001
Distance	0.000
Aquaculture	0.000
Height: Species	0.000
River mouth: Distance	0.000
Species: River mouth	0.000
Species: Distance	0.000
River mouth: Aquaculture	0.000
Distance: Aquaculture	0.318
Species: Aquaculture	0.000
Species: River mouth: Distance	0.999
River mouth: Distance: Aquaculture	0.977
Species: River mouth: Aquaculture	0.000
Species: Distance: Aquaculture	0.332
Species: River mouth: Distance: Aquaculture	0.000
