# Supplementary figures and images for: Defoliation in mangrove saplings vary depending on species and environment
Source: Biodivers Data J. 2025 May 23;13:e140659. doi: 10.3897/BDJ.13.e140659 (PMC12125597; doi:10.3897/BDJ.13.e140659)

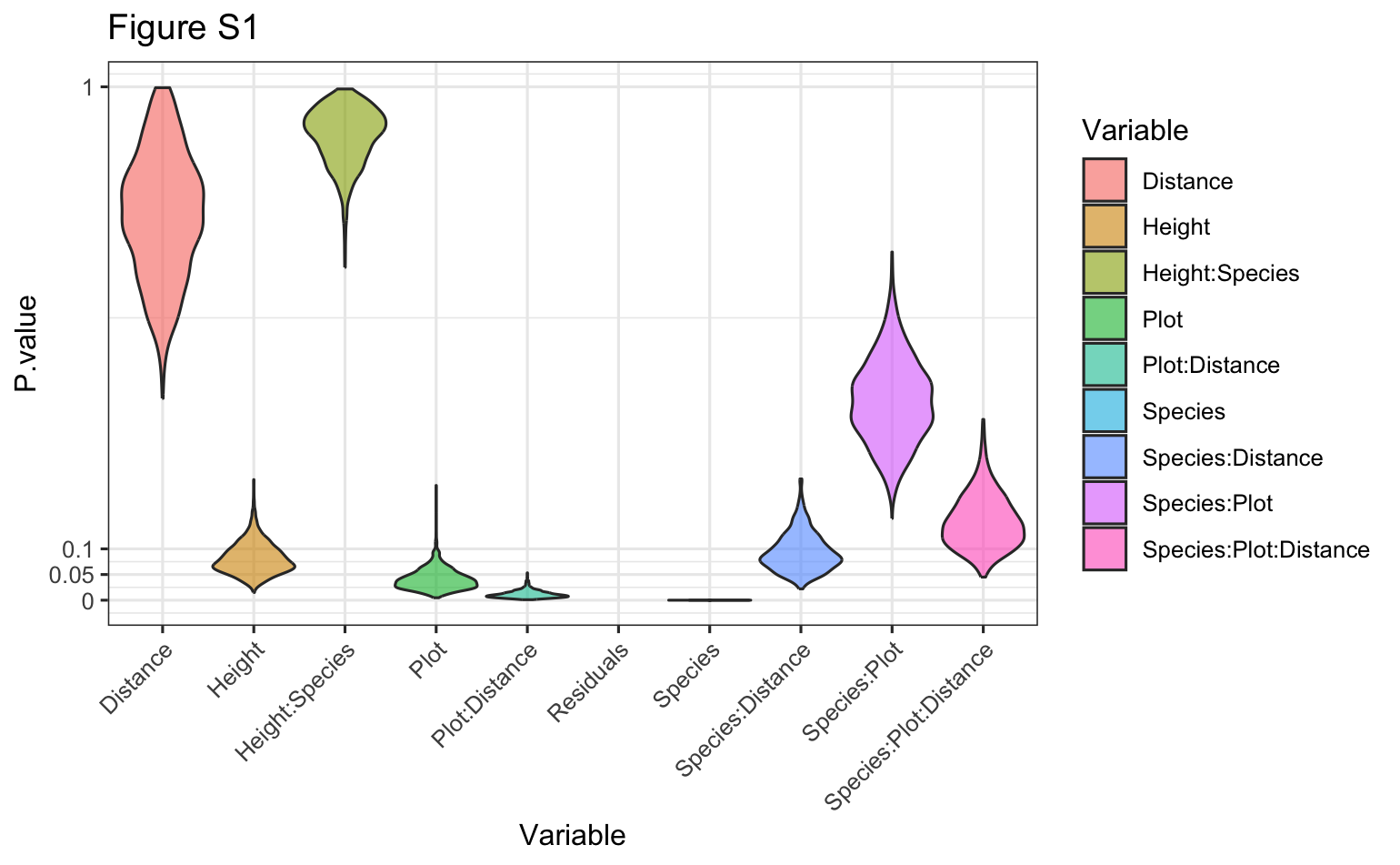

Supplement: Supplementary material 1 — Figure S1 [file bdj-13-e140659-s001.png]

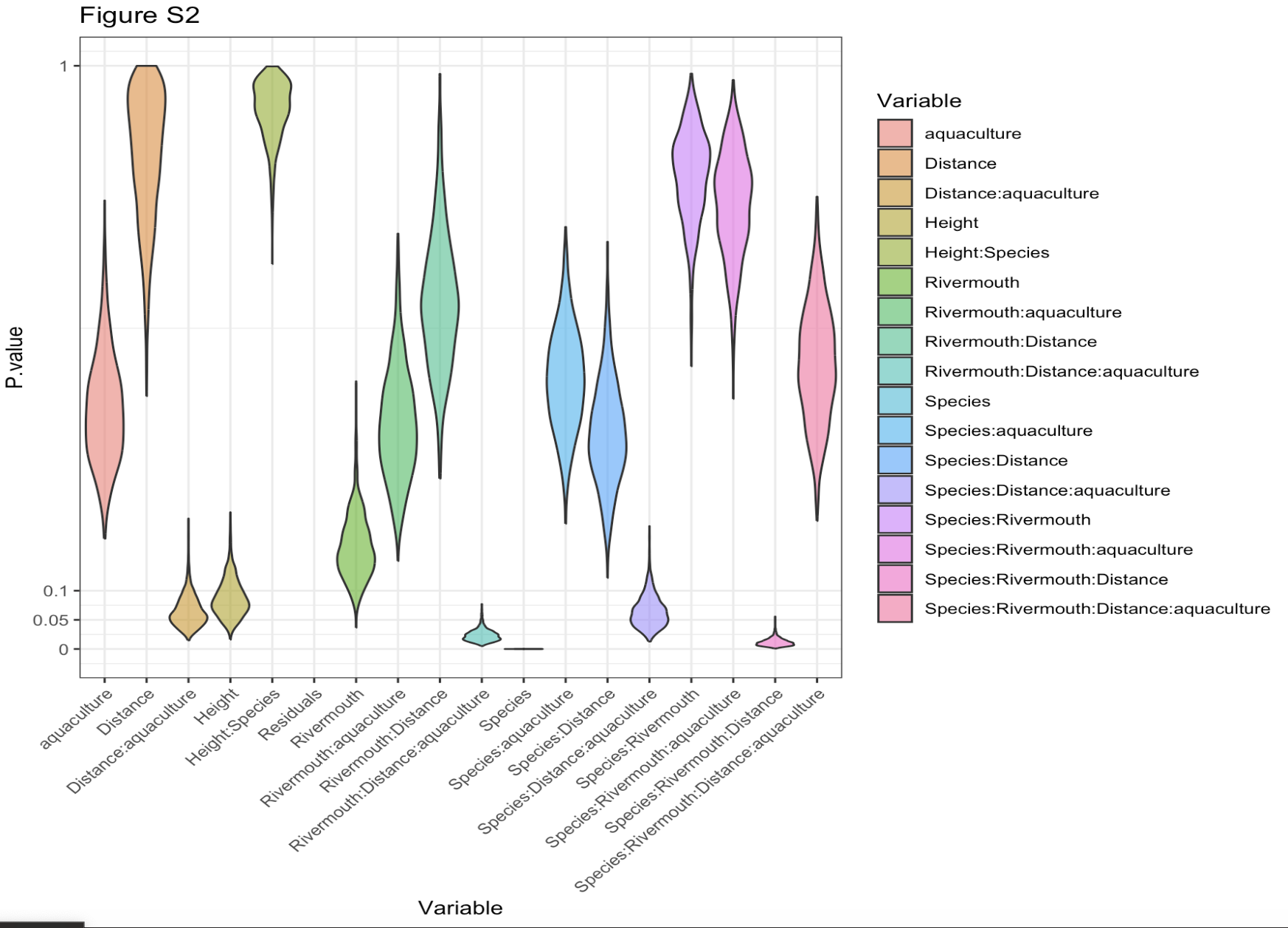

Supplement: Supplementary material 2 — Figure S2 [file bdj-13-e140659-s002.png]
