# Supplementary material for: Defoliation in mangrove saplings vary depending on species and environment
Source: Biodivers Data J. 2025 May 23;13:e140659. doi: 10.3897/BDJ.13.e140659 (PMC12125597; doi:10.3897/BDJ.13.e140659)
Supplement: Supplementary material 3 — Table S1 [file bdj-13-e140659-s003.docx]

| Table S1. Coefficient estimates and standard errors (to 2 d.p.) | | |
| --- | --- | --- |
| Variable name | Estimate | Standard Error |
| Species BRGY | -20.78 | 1.96 |
| Species RHAP | 88.39 | 11.09 |
| Species RHMU | -25.76 | 1.96 |
| Site 2 | 63.99 | 9.41 |
| Site 3 | 114.91 | 9.77 |
| Site 4 | 73.99 | 9.41 |
| Site 5 | -23.53 | 16.24 |
| Site 6 | 72.76 | 9.48 |
| Site 7 | 43.32 | 10.36 |
| Site 8 | 76.04 | 9.40 |
| Site 9 | 67.80 | 9.44 |
| Site 10 | 84.08 | 9.59 |
| Site 2: Distance | -0.17 | 0.02 |
| Site 3: Distance | 0.05 | 0.02 |
| Site 4: Distance | -0.28 | 0.02 |
| Site 5: Distance | -0.17 | 0.04 |
| Site 6: Distance | 0.05 | 0.04 |
| Site 7: Distance | -0.09 | 0.02 |
| Site 8: Distance | -0.22 | 0.02 |
| Site 9: Distance | -0.15 | 0.03 |
| Site 10: Distance | -0.19 | 0.02 |
