# Supplementary material for: Defoliation in mangrove saplings vary depending on species and environment
Source: Biodivers Data J. 2025 May 23;13:e140659. doi: 10.3897/BDJ.13.e140659 (PMC12125597; doi:10.3897/BDJ.13.e140659)
Supplement: Supplementary material 4 — Table S2 [file bdj-13-e140659-s004.docx]

| Table S2. Coefficient estimates and standard errors (to 2 d. p.) | | |
| --- | --- | --- |
| Variable name | Estimate | Standard error |
| Species BRGY | -6.84 | 0.43 |
| Species RHAP | -4.93 | 0.41 |
| Species RHMU | 0.21 | 2.24 |
| Species BRGY: River mouth: Distance | -0.03 | 0.01 |
| Species RHAP: River mouth: Distance | -0.01 | 0.01 |
| Species RHMU: River mouth: Distance | -0.13 | 0.03 |
| River mouth: Distance: Aquaculture | -0.11 | 0.01 |
